# Voice-Enabled Intelligent Virtual Agents for People With Amnesia: Systematic Review

**DOI:** 10.2196/32473

**Published:** 2022-04-25

**Authors:** Roel Boumans, Yana van de Sande, Serge Thill, Tibor Bosse

**Affiliations:** 1 Behavioural Science Institute Radboud University Nijmegen Netherlands; 2 Donders Institute for Brain, Cognition, and Behaviour Radboud University Nijmegen Netherlands

**Keywords:** intelligent virtual agent, amnesia, dementia, Alzheimer, systematic review, mobile phone

## Abstract

**Background:**

Older adults often have increasing memory problems (amnesia), and approximately 50 million people worldwide have dementia. This syndrome gradually affects a patient over a period of 10-20 years. Intelligent virtual agents may support people with amnesia.

**Objective:**

This study aims to identify state-of-the-art experimental studies with virtual agents on a screen capable of verbal dialogues with a target group of older adults with amnesia.

**Methods:**

We conducted a systematic search of PubMed, SCOPUS, Microsoft Academic, Google Scholar, Web of Science, and CrossRef on virtual agent and amnesia on papers that describe such experiments. Search criteria were (*Virtual Agent* OR *Virtual Assistant* OR *Virtual Human* OR *Conversational Agent* OR *Virtual Coach* OR *Chatbot*) AND (*Amnesia* OR *Dementia* OR *Alzheimer* OR *Mild Cognitive Impairment*). Risk of bias was evaluated using the QualSyst tool (University of Alberta), which scores 14 study quality items. Eligible studies are reported in a table including country, study design type, target sample size, controls, study aims, experiment population, intervention details, results, and an image of the agent.

**Results:**

A total of 8 studies was included in this meta-analysis. The average number of participants in the studies was 20 (SD 12). The verbal interactions were generally short. The usability was generally reported to be positive. The human utterance was seen in 7 (88%) out of 8 studies based on short words or phrases that were predefined in the agent’s speech recognition algorithm. The average study quality score was 0.69 (SD 0.08) on a scale of 0 to 1.

**Conclusions:**

The number of experimental studies on talking about virtual agents that support people with memory problems is still small. The details on the verbal interaction are limited, which makes it difficult to assess the quality of the interaction and the possible effects of confounding parameters. In addition, the derivation of the aggregated data was difficult. Further research with extended and prolonged dialogues is required.

## Introduction

### Background

Older adults often complain about amnesia or increasing memory problems, although these cognitive changes affect some individuals more than others [1]. Although some degree of cognitive slowing is typical of normal aging, when the acquired cognitive impairment has become severe enough to compromise social or occupational functioning, the diagnosis of dementia is typically established [2]. Alzheimer disease (AD) is the most commonly diagnosed form of dementia. If the functional abilities of patients are still essentially preserved while their cognitive abilities are in between those associated with normal aging and dementia, people are typically diagnosed with mild cognitive impairment [2].

This gradation in cognitive abilities does not suggest a necessary sequence of normal cognitive slowing to mild cognitive impairment to dementia; most people will only experience normal cognitive slowing upon aging, some will develop mild cognitive impairment and some will develop dementia. For the latter category, the period between first serious cognitive complaints and the diagnosis of dementia can be 10 years and depends on many factors such as age, sex, and general physical premorbid condition [3]. The duration of survival after AD diagnosis is 10.2 years for men aged 65 years and 13.2- years for women of the same age [4]. In this period after diagnosis of AD, the period for need for home care typically lasts 3.7-4.7 years and for institutional care, 2.2-3.2 years [4].

During the period of normal cognitive decline without the need for additional care, people may benefit from personalized support. In follow-up phases, where people need extended home care or institutional care, the need for personalized support increases. In the early phases, such support is often provided by partners, children, friends, or other relatives. When these are not available, but the patient is still living independently at home, professional help by, for example, case managers, district nurses, and meal delivery services may be needed upon the indication of a patient’s general practitioner. If the need for institutional care arrives, people may move to a nursing home and be cared for by nurses and other health care professionals. However, in each of these phases, informal caregivers or health care professionals are often not sufficiently available for the needs of the patient. For example, Buchan et al [5] have identified a global shortage of 17 million health care workers in 2019. This shortage will only increase owing to the growing percentage of older adults in the total population (9% in 2019 and 12% in 2030) [6]. Hence, society is looking for alternative solutions such as technological systems that could support health care professionals in their work by taking over automatable tasks. One such potential solution can be offered by intelligent virtual agents (IVAs).

IVAs can be defined as interactive digital characters that exhibit human-like qualities and can communicate with humans using natural human modalities such as facial expressions, speech, and gestures [7]. This broad definition includes intelligent virtual characters that manifest themselves as text-based chatbots on smartphone or tablet and virtual characters in the form of a human head or a complete person on a tablet or computer screen. Several other terms are used for this type of agent, such as virtual assistant, (embodied) conversational agent, cognitive assistant, chatbot, intelligent assistive technology, and virtual human. IVAs have also been the subject for a series of conferences on this theme organized by the Association for Computing Machinery since 1998 [7]. As it has been a topic of research for more than 3 decades now for many applications, a large volume of research papers have been published on this subject.

For the purpose of this study, the main interest is in IVAs implemented as onscreen virtual characters that support older adults through autonomous verbal interaction. This specification is based on 3 reasons. First, from the perspective of the patient, verbal interaction is considered the easiest and most natural way to communicate and build rapport with an agent [8]. Second, a virtual character is more inviting to have a verbal communication with than a text-based chatbot. Third, once a person is used to this type of communication, a many-year support period during various forms of slowly progressing cognitive decline could be possible: talking is something people can do for a long period, whereas pushing buttons on a touchscreen still assumes some digital literacy, and this may disappear with cognitive decline [9].

IVAs have potential advantages for organizations that deliver home or institutional care. These organizations could decrease the need for a 24×7 human support team if a large part of the frequently asked support questions can be handled through an intelligent dialogue with an IVA. Furthermore, IVAs are immediately available and there is no need to wait in line for the availability of a health care organization employee. Interaction data can be stored and analyzed both on a personal and on an aggregated level, where the latter is of interest to the health care organization as well as to governmental health care control institutions. Obviously, ethical and privacy concerns need careful consideration and as a start can be addressed by a compliance check with the ethics guidelines for trustworthy artificial intelligence (AI), as published by the European Commission [10,11]. At the same time, IVAs have not yet been widely introduced in the consumer market and can thus far be found primarily in laboratory environments. Thus, the question arises as to whether IVAs have actually been developed and evaluated as verbal coaches or companions for cognitively impaired older adults. Therefore, this research first considered related systematic reviews and then identified the current state of the art through a systematic literature review.

### Other Reviews

Other systematic reviews on virtual agents in health care revealed that the number of experiments with voice-enabled agents was rather limited. Xie et al [12] conducted a review on AI specifically for caregivers of persons with dementia, but did not report on any virtual agents for which an actual experiment had been conducted. Schachner et al [13] reviewed papers on AI-based conversational agents for chronic conditions. Although they found 2052 articles, only 10 met their inclusion criteria for research on chronic diseases involving an AI-based conversational agent. Of these 10, 2 papers dealt with dementia, but did not include a virtual agent.

Ienca et al [14] performed a systematic review on intelligent assistive technology for several forms of dementia including AD and included 571 studies. They examined the technological type of the interventions among others, but did not make a clear distinction on whether the intervention involved a virtual agent capable of 2-way verbal communication.

Bevilacqua et al [15] conducted a systematic review of the effectiveness of coaching through technology among older adults. From their original set of 2186 articles, 8 met their inclusion criteria, among which the criterion of the study was a randomized controlled trial. This criterion was more stringent than the ones used in this study, meaning that studies that could be included in this study might be excluded in their search strategy. In contrast, none of the studies they found aimed at people with cognitive problems.

**Figure 1:**
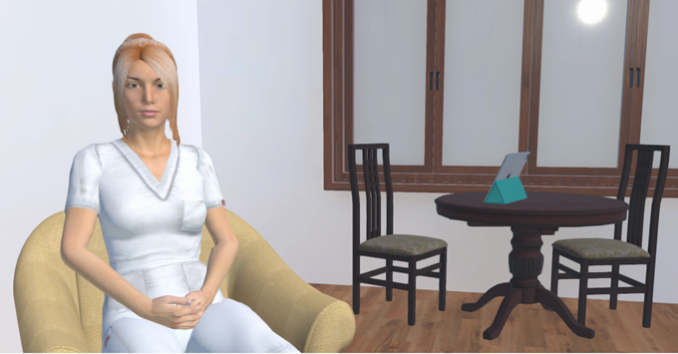
A virtual agent under development at the Behavioural Science Institute.

Car et al [16] conducted a scoping review on conversational agents in health care. They did not report on any virtual agents such as those depicted in Figure 1, as their results were restricted to smartphone apps. Laranjo et al [17] also conducted a systematic review on conversational agents in health care. From the initial 1513 search results, they included 17 studies that evaluated 14 different conversational agents with unconstrained natural language input capabilities. From their results, 2 studies focused on an embodied conversational agent similar to that in Figure 1, but one of these studies was aimed at military personnel with posttraumatic stress syndrome (PTSS) and the other one, on training for people with autism spectrum disorders.

King and Dwan [18] created an inventory of electronic memory aids for people with dementia who experience memory loss increasing with age. They found 16 studies that met their inclusion criteria, one of which also met the criteria drawn up for this study: a study by Tokunaga et al [19] on a memory-aid service agent.

Provoost et al [20] reviewed embodied conversation agents in clinical psychology. They included 54 publications after an initial search result of 1117 references, but the disorders studied were autism, depression, anxiety, PTSS, schizophrenia, and substance abuse. Depression and anxiety are often associated with dementia. However, the studies related to these subgroups did not meet the criterion of a virtual agent.

Loveys et al [21] specifically examined the design features of embodied conversational agents and the extent to which these have an effect on the relationship quality between human and agent. Their systematic review resulted in 43 studies that examined design features such as language use, behavior, emotional expression, embodiment, appearance, personality, environment, and a combination of these. However, none of these studies were aimed at people with dementia.

Milne-Ives et al [22] performed a recent systematic review on the effectiveness of AI conversational agents in health care. They found 31 studies including a variety of conversational agents, among which were 14 chatbots and 6 embodied conversational agents; however, none specifically targeted people with cognitive problems such as dementia.

It is possible that some of the papers that were excluded from the aforementioned reviews met the inclusion criteria for this study. In any case, there is a gap when it comes to IVAs for people with amnesia, dementia, or AD, and with whom 2-way verbal interaction experiments have been conducted. This gap calls for a specific systematic review on that topic.

### Objectives

This study aimed to evaluate IVAs and their interaction functionality, which have demonstrated a verbal communication for people with amnesia. Our hypothesis is that IVAs designed to assist older adults with memory problems have a positive usability. This review reports on studies with various voice-based IVA applications that have been developed to support these patients and the studies, which included (pilot) evaluations with this target group.

## Methods

### Eligibility Criteria

For the purpose of this review, an IVA was defined as an agent with a virtual embodiment (full body or face only) that was capable of *speaking* to a human (ie, playing a generated audio file). The agent shall be capable of having a 2-way verbal dialogue, that is, the human could speak to the agent, the agent could process the human input, and as a result, the agent would speak to the human. Therefore, the agent should also be capable of speech recognition, that is, converting the human audio signal (human utterance) into a text string. This text string should then be made available for response determination or natural language understanding, that is, intent, belief, or desire determination and action selection. Verbal response actions should be input for a text-to-speech function. The agent should be displayed on a permanently available screen and should not require the mounting of a virtual reality headset that is difficult to wear in a 24×7 setting and may also cause motion sickness and disorientation [23].

Studies eligible for review were further required to (1) include an experiment, pilot study, or randomized controlled trial with experimental results, thus not describing only requirements or designs; (2) target participants from an older adult population with potentially memory-related problems; and (3) be published in a peer-reviewed journal or in peer-reviewed conference proceedings.

Studies were excluded if (1) the agent was a physical robot, a purely text-based chatbot, or a virtual reality or augmented reality character; (2) the study concerned a Wizard of Oz study in which the researcher mimicked the agent and circumvented automated speech recognition and natural language processing (NLP) challenges; (3) the paper language was other than English; (4) the search result concerned a thesis or dissertation (as far as these were not peer reviewed and therefore did not meet publishable standards); (5) the search result was an abstract only, a PowerPoint, or a website; and (6) the full text was not available for the authors.

### Search Strategy

A systematic search was conducted for keywords on papers included in electronic databases from health and computer sciences including PubMed, SCOPUS, Microsoft Academic, Google Scholar, Web of Science, and CrossRef. The search term was (*Virtual Agent* OR *Virtual Assistant* OR *Virtual Human* OR *Conversational Agent* OR *Virtual Coach* OR *Chatbot*) AND (*Amnesia* OR *Dementia* OR *Alzheimer* OR *Mild Cognitive Impairment*) and the search period was 2010 to present. This period has been defined for several reasons. First, the statement by Wargnier et al [24] that they did not find a publication on a usability interaction of a talking virtual agent with older adults with cognitive impairment. Second, 2010 was named as the start of the era of deep learning in speech recognition, which caused an explosion in the success of speech recognition applications [25,26]. Third, the limited number of relevant papers originating before 2010 and found in other systematic reviews [12-16,18,20-22]. The Publish or Perish tool was used to conduct the search, collect the results, and export them via csv-files to an Excel spreadsheet for study selection [27].

### Study Selection

Two reviewers (RB and YvdS) independently conducted the search and compared and agreed on the results. Titles were screened upon clearly including the words of the search or being strongly related, based on the assumption that the title of the research paper should be clear on its contents. In case of doubt, the abstract was consulted. From this selection, the abstracts were reviewed for a second selection. The abstracts should include a reference to a verbal interaction experiment of a virtual agent with older adults and should be published in a peer-reviewed journal paper or conference proceedings. Abstracts that met these criteria were subsequently discussed and selected for full-paper text analysis. Studies deemed eligible for review were included in data synthesis.

### Data Collection

The data of the selected papers are provided in a table format. The top row includes the authors, title of the paper, journal or conference proceedings where it was published, and the year of publication. Row 2, column 1 describes the data on the study: country, study design type, target sample size, controls, and study aims. In row 2, column 2, the actual experimental population is reported, whereas row 2 and column 3 details the intervention. Row 3, column 1 specifies the typical interaction between the IVA and the human, with specific attention to the verbal interaction options for the human and the NLP techniques that were applied. Row 3, column 2 provides a summary of the reported results, and row 3, column 3 presents an image of the IVA.

### Quality of Study Evaluation

The standard quality assessment criteria for evaluating primary research papers developed for qualitative research by Kmet et al [28] were used by reviewers TB and ST to evaluate the quality of the studies found and the risk for bias. This method uses the checklist presented in Textbox 1.

The eligible papers were scored for the aforementioned items as yes (2 points), partial (1 point), no (0 points), or not applicable. Agreement was reached on whether an item could be scored or defined as *not applicable* and ignorable. The score of the reviewers TB and ST per item was averaged. The overall score per paper was calculated by dividing the summed score by the total number of scored items, multiplied by 2. This resulted in a score between 0 and 1.

**Standard quality assessment criteria.**

**Checklist used**

- Question or objective sufficiently described?
- Study design evident and appropriate?
- Method of participant or comparison group selection or source of information or input variables described and appropriate
- Participant and comparison group, if applicable, characteristics sufficiently described?
- If interventional and random allocation was possible, was it described?
- If interventional and blinding of investigators was possible, was it reported?
- If interventional and blinding of participants was possible, was it reported?
- Outcome and (if applicable) exposure measures well defined and robust to measurement or misclassification bias? Means of assessment reported?
- Sample size appropriate?
- Analytic methods described or justified and appropriate?
- Some estimate of variance is reported for the main results?
- Controlled for confounding?
- Results reported in sufficient detail?
- Conclusions supported by the results?

## Results

### Study Selection

**Figure 2:**
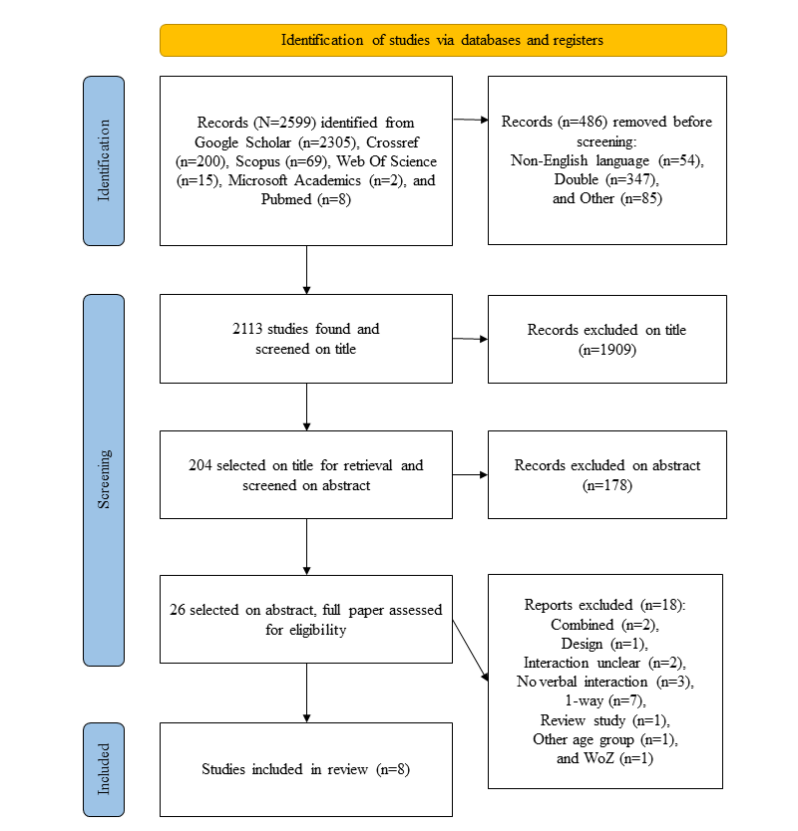
Study flow.

The search was conducted in the first week of March, 2021. In total, 2599 papers were found, and the study flow in accordance with the PRISMA (Preferred Reporting Items for Systematic Reviews and Meta-Analyses) methodology can be found in Figure 2 [29]. Most of the papers (2305/2599, 88.69%) found by the reviewers YvdS and RB were obtained from Google Scholar search. A total of 486 records were removed before screening, because they were published in a different language (n=54, 11.1%), they appeared more than once in the list (sometimes under a slightly different title; n=347, 71.4%), or for other reasons (n=85, 17.5%) such as not showing an author or having an illegible title. The resulting 2113 papers were screened on the text of the title and papers that did not clearly mention one of the search terms or a synonym in the title were excluded. In case of doubt, the abstract was consulted. This resulted in 204 papers that were subsequently screened by the reviewers for their abstracts. After screening abstracts, 26 papers were selected for full-text analysis. After full-text analysis, 8 papers that satisfied all the criteria were included in this review. Multimedia Appendix 1 [29] and Multimedia Appendix 2 [29] present the PRISMA checklists.

### Selected Studies

This review resulted in 8 selected studies. The study data are summarized in Table 1 and sorted by avatar. The average number of participants in the studies was 20 (SD 12).

**Table 1:** Selected studies.

Authors and title	Study design	Experiment population	Intervention	Human-agent interaction	Results			Agent image	
Ali R et al [30]. Aging and engaging: A pilot randomized controlled trial of an online conversational skills coach for older adults.	Country: United States; study design: pilot randomized controlled trial; N=18; intervention and control group; study aim: to assess feasibility and acceptability of the VA^a^	N=18, 1 lost to follow-up; aged >60 years; having self-reported mild communication difficulties with social skills that could be attributable to memory problems	In the intervention group, the VA had weekly sessions with each participant, including three 2- to 3-minute open dialogues with the participant on a selection of general topics (eg, weather, pets, retirement, life goals, growing older, and spirituality). The control group was provided with videos.	The VA was a web-based application using computer system including camera and microphone from the participant’s home to record participant utterances and expressions. Open dialogue included ASR^b^ and TTS^c^. The ASR used a hierarchical tree to classify participant utterances and determine responses.	The system usability score was 69.5 on a 0-100 scale, where 68 was considered as “good” usability. Participants randomized to the VA demonstrated significantly fewer impairments in nonverbal communication at follow-up compared with the control group, whereas the results were nonsignificant for participants with verbal impairment.			Figure 3	
Razavi et al [31]. Dialogue design and management for multisession casual conversation with older adults. Precursor study to Ali R et al [30].	Country: United States; N=8. Evaluative study aimed at older adults where each participant had a 10- to 20-minute conversation with the VA. The system was designed for geriatric patients and with input from gerontologists. Study aim: examination of conversation quality.	N=8; older adults. No details given.	Have a short conversation on several topics, categorized as easy, medium, and hard. They covered 30 themes among which were hobbies, weather, cooking, life goals, and spirituality. Average duration was a few minutes with 3-5 turns; no details given.	A 2-way interaction; VA asks question or reacts on participant response [1].	Participants were asked to score 4 variables on a 5-point scale from strongly disagree (1) to strongly agree (5). Ease of use was scored as 4.3, learnability as 3.9, confidence in using as 4.3, and user-friendliness as 4.6.			Figure 4	
Wargnier et al [24]. Usability assessment of interaction management support in LOUISE^d^, an ECA^e^-based user interface for elders with cognitive impairment.	Country: France; feasibility study; N=14; no control group. Study aim was usability assessment of the LOUISE system.	N=14; aged >65 years; diagnosed with cognitive impairment	VA asked participant to perform 4 tasks: drink water, take a pill, measure blood pressure, and select meal. Participants could choose between 2 VA embodiments: “Louise” (left image) and “Charlotte” (right image).	The VA questions were provided verbally. Participants could verbally answer with “yes” or “no.” Microsoft Speech ASR was used.		All but one participant could interact with LOUISE. Of the 14 participants, 11 completed the 4 scenarios, but from these 11 situations, only 1 was conducted “in WoZ mode,” and 1 showed sensor failures. Thus, ultimately data from 9 participants were available. Participants often forgot they could only say “yes” or “no.” ASR error rate was 20%.			Figure 5
Tokunaga et al [32]. Virtual caregiver: Personalized smart elderly care.	Country: Japan; feasibility study; N=11; no controls.	N=11; 9 women; older inhabitants of the daycare center	VA conducted greeting, confirmation of basic personal information, quiz, and playing music. Measurement was done using a 10-question usability score between 1 and 4. Design of the avatar was similar to Tokunaga et al [19].	The VA makes statements and asks questions. The paper does not provide details on the user input types. No description of NLP^f^ function was given.		Average usability score was 3.58. Participants found playing music as especially useful.			Figure 6
Tokunaga et al [19]. Implementation and evaluation of interactive memory-aid agent service for people with dementia. Related to Tokunaga et al [32].	Country: Japan; exploratory study; N=17. Study aim was to confirm that the patients could interact with the agent service using some interactions (eg, voice or touch).	N=17; older adult patients, aged 46-84 years, 12 women. Mean MMSE^g^ 22.9, meaning some cognitive impairment.	A nondetailed scenario in which participant had to perform certain tasks upon verbal instruction of the VA. The participant could respond by voice or by touch button.	VA asks questions, and the participant reacts. No details were provided. NLP characteristics were unclear.			Participants did not always hear the agent because of hearing impairments or microphone quality. Touch button operation was difficult for the older adults not accustomed to smartphones or tablets. VA did hear the patient only after a second utterance. Patients were sometimes surprised and did not know what to do if the system did not react as expected.		Figure 7
Tsiourti et al [33]. A virtual assistive companion for older adults: design implications for a real-world application.	Countries: Switzerland, Portugal, and the Netherlands; N=20; design: longitudinal evaluation study; goals: to examine empirically interaction with ECA at home and explore ECA acceptance, perceived usability, and usefulness.	A total of 24 older adults living at home with average age of 77.9 years in 2 countries: the Netherlands (N=11) and Switzerland (N=13). In Netherlands, the number of dropouts was 4.	In the Netherlands, researchers visited participants for joint sessions with the VA 2-3 times a week. System user options were tried in no specific order or method but included reminders and memory programs. Detailed use scenarios on Switzerland were unclear, participants seemed to use the system autonomously.	The users interacted with the companion using a multimodal interface including automatic speech recognition and a graphical touch-based user interface menu (messages and agenda). ASR used Kinect for Windows SDK^h^ to perform speech recognition for predefined speech commands that the users had to remember.			Empirical findings were problems with speech recognitions, remembering of user interaction options by participants, and the nonintuitiveness of the user interface. Acceptance was “well received.” Usability was 62.2 for participants from Switzerland and 52 for those from the Netherlands on an unspecified scale. Usefulness in participants from the Netherlands was not reported and in participants from Switzerland as 2.3-2.5 on a scale of 0 to 5.		Figure 8
Jegundo et al [34]. Perceived usefulness, satisfaction, ease of use, and potential of a virtual companion to support the care provision for older adults.	Country: Portugal; observational study; N=46.	Target group was older adults needing formal care. Convenience sample of older adults in daycare centers; 34 women, 12 men; mean age 63.6 (SD 20.5) years.	All interactions were by touch; only interaction on “News” was by voice. Sessions were all witnessed by researchers.	VA responded verbally by “News” commands (“Open news” or “Read news”). All other interactions were through touch buttons (play game or show agenda).			CaMeLi^i^ presents a good degree of usefulness, satisfaction, and ease of use. CaMeLi was a barrier to 11 participants and a facilitator for 35 participants.		Figure 9
Oliveira et al [35]. A multiplayer voice-enabled game platform for the elderly.	Country: Portugal; design: quiz game with VA as host; n=21; no control group; study aim: feasibility	A total of 2 groups: “Young” (aged 24-28 years; n=4) and “Elder” (aged 59-88 years; n=17); divided into 2 subgroups: “tested at home” (n=9) and “tested at the senior university” (n=8). Target group comprised people with dementia, but cognitive status of participating older adults was not described.	Participants had to answer quiz questions by a web-based quiz host. Answers could be open answers.	ASR and TTS were developed at the university laboratory. A 2-way spoken question and answer was used. VA used was a passive cartoon.		Participants enjoyed the game and were stimulated to interact with each other.			Figure 10

^a^VA: virtual agent.

^b^ASR: automated speech recognition.

^c^TTS: text-to-speech.

^d^LOUISE: Lovely User Interface for Servicing Elders.

^e^ECA: embodied conversational agent.

^f^NLP: natural language processing.

^g^MMSE: Mini Mental State Examination.

^h^SDK: software development kit.

^i^CaMeLi: Care Me for Life.

**Figure 3:**
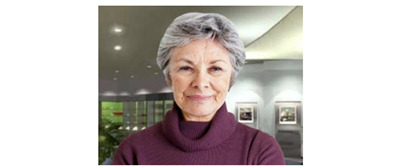
Agent by Ali et al [30].

**Figure 4:**
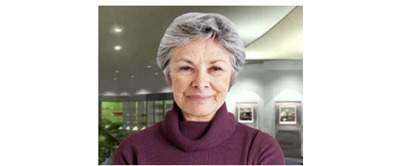
Agent LISSA (Live Interactive Social Skills Assistance) by Razavi et al [31].

**Figure 5:**
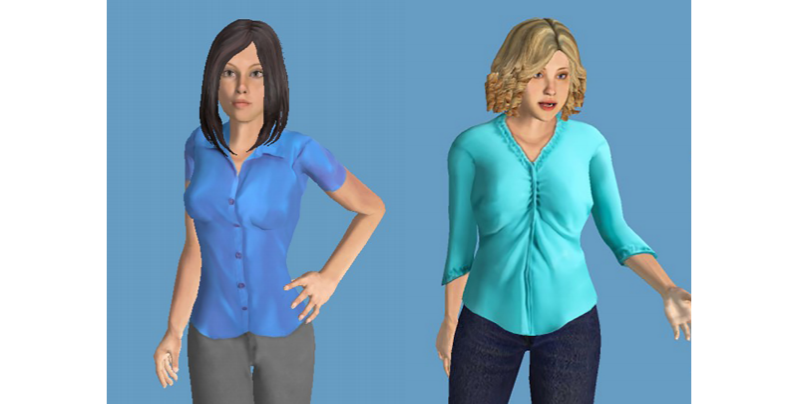
Agent Louise (left) and Charlotte (right) by Wargnier et al [24].

**Figure 6:**
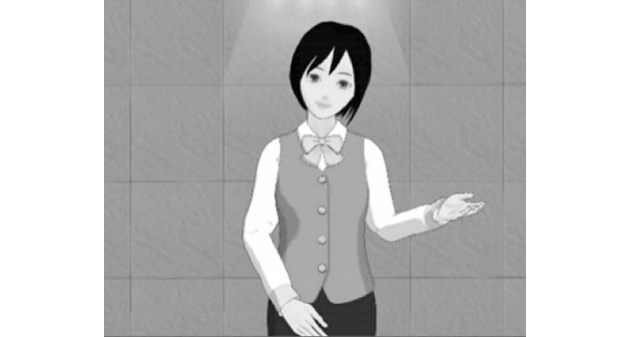
Virtual caregiver by Tokunaga et al [32].

**Figure 7:**
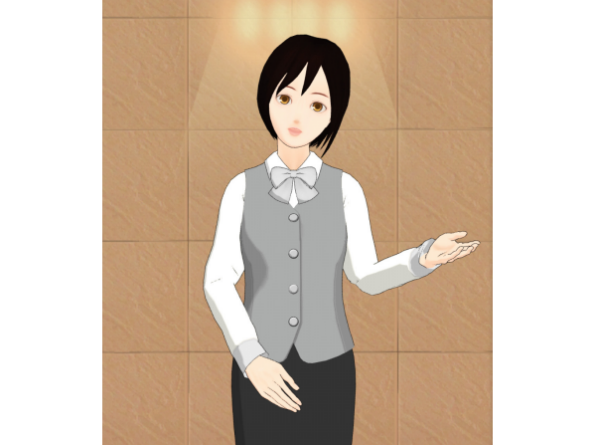
Agent by Tokunaga et al [32].

**Figure 8:**
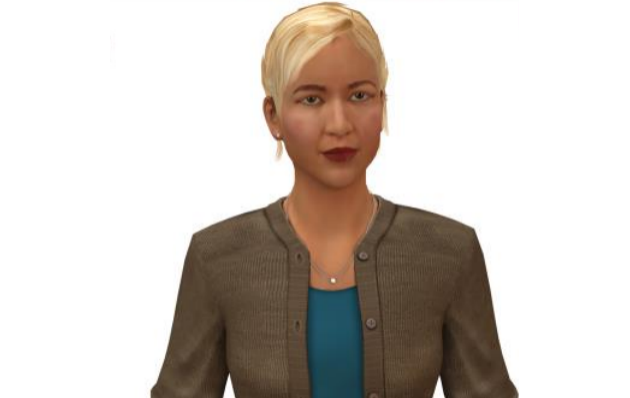
Agent Mary by Tsiourti et al [33].

**Figure 9:**
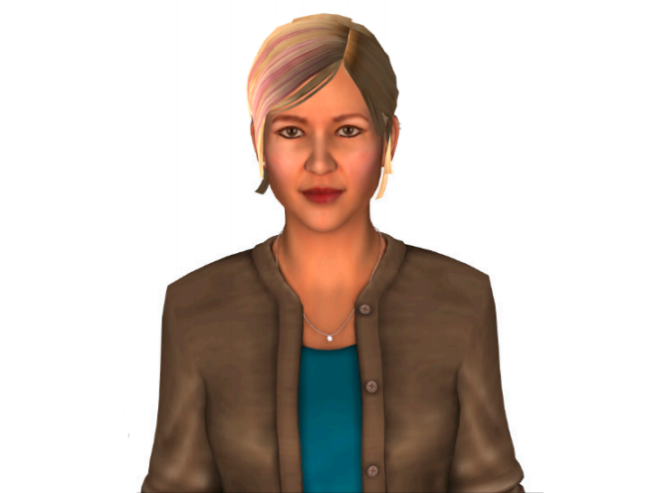
Agent by Jegundo et al [34].

**Figure 10:**
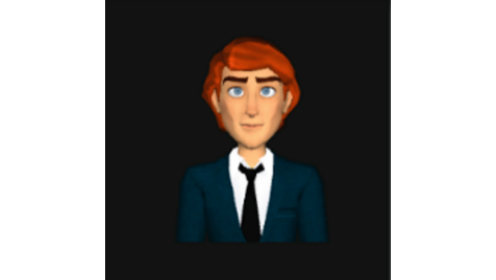
Agent by Oliveira et al [35].

A total of 3 studies might have been included but on detailed consideration have been excluded. Parsons et al [36] reported on a human avatar that is portrayed as a physician and conducts a neuropsychological assessment. However, this was not explicitly aimed at older adults with memory problems, and the verbal interaction description lacked details to judge the dialogue form. In all, 2 studies reported the recording of human utterances in response to an avatar interview question, which were subsequently analyzed offline on the prevalence of cognitive impairment [37,38]. Therefore, they were not considered real dialogue.

### Study Quality Evaluation

The results of the quality assessment are presented in Table 2. The average quality score was 0.69 (SD 0.08).

**Table 2:** Scores of methodological quality assessment of the included studies.

	Ali et al [30]	Razavi et al [31]	Wargnier et al [24]	Tokunaga et al [32]	Tokunaga et al [19]	Tsiourti et al [33]	Jegundo et al [34]	Oliveira et al [35]
Objectives	2	1.5	1	1	1	1	1.5	1.5
Study design	1.5	1	1.5	1.5	1.5	2	2	1.5
Method	2	1.5	1	0.5	0.5	1	1.5	1.5
Participants	2	0.5	1.5	1	1.5	1	1.5	1.5
Random allocation	1	N/A^a^	N/A	N/A	N/A	N/A	N/A	N/A
Blinding investigators	2	N/A	N/A	N/A	N/A	N/A	N/A	N/A
Blinding participants	0	N/A	N/A	N/A	N/A	N/A	N/A	N/A
Outcomes	1.5	2	0.5	2	1	1.5	1.5	0.5
Sample size	1	1.5	1.5	1	1.5	1.5	1.5	1.5
Analytic methods	1	N/A	N/A	N/A	1.5	N/A	1.5	2
Variance estimates	2	2	1	1	N/A	N/A	2	1
Confounding controls	1.5	N/A	N/A	N/A	N/A	N/A	N/A	N/A
Results reporting	1.5	2	1.5	2	2	1	1.5	1
Conclusions	1.5	1.5	1	1	1	1.5	2	1.5
Total	20.5	13.5	10.5	11	11.5	10.5	16.5	13.5
Maximum score	28	18	18	18	18	16	20	20
Summary score	0.73	0.75	0.58	0.61	0.64	0.66	0.83	0.68

^a^N/A: not applicable.

## Discussion

### Principal Findings

Despite the fact that IVAs have been a topic of study since 1998, the number of studies that actually show a 2-way verbal interaction with older adults with amnesia is relatively low, and only 8 studies were found. One of the main bottlenecks is the quality of the speech-to-text function. For example, Sidner et al [39] described this function as *a technical challenge*, and therefore chose touch buttons on a screen as human input means to the virtual agent. Several other studies followed the same approach and were not included in this review.

In the studies found, it was particularly difficult to assess participants’ perception of verbal interaction. In 4 studies, the user could only give yes or no or a few other short commands [24,33,34,40]. The user verbal command options in the studies by Tokunaga et al [19,32] were unclear. Only the studies by Ali et al [30] and Razavi et al [31] report a short (3-5 turns) open dialogue on a range of topics such as *weather, pets, retirement, life goals, growing older, and spirituality.* A total of 9 studies also provided little information on their NLP pipeline. Ali et al [30] and Razavi et al [31] designed a pattern-matching solution based on *gist-clauses*, which are a combination of the IVA question and the answer received. Wargnier et al [24] and Tsiourti et al [33] used Microsoft speech recognition components for speech recognition, and the utterance text strings were matched with predefined commands. Oliveira et al [35] used an in-house developed speech recognition subsystem. In addition to this information, a few other details are provided that allow us to compare the advantages and disadvantages. The other 3 studies did not provide any information regarding the NLP pipeline. However, all 8 studies reported a generally positive attitude of the participants toward the agent. Video recordings would have been helpful in assessing the details of human-agent interaction.

The main qualitative outcome reported in these studies was the usability of the system. This usability was measured differently between the studies, and the methods and data are provided in Table 3. Ali et al [30] and Jegundo et al [34] used the well-known System Usability Scale [40]. Wargnier et al [24] and Tsiourti et al [33] did not use existing usability questionnaires from the literature, such as the System Usability Scale, but created new usability questionnaires. The studies by Tokunaga et al [19,32] were more oriented toward the functional performance of the agent, whereas Oliveira et al [35] did not report clear usability measurement methods and results. A comparison between the usabilities is difficult to make because of the difference in the methods and scales used. The difference in scales also does not allow us to calculate an aggregated mean value of the usability for the studies combined. Furthermore, the reason for the relatively low number of participants (*µ*=20) is probably the explorative or feasibility assessment character of the studies found. A system usability study should preferably include between 20 and 30 participants [40]. Our hypothesis that IVAs designed to assist older adults with memory problems have a positive usability, given the data from the table, indicates that there is reason to believe that it is true.

**Table 3:** Comparison between usability scores.

Reference	Usability
Ali et al [30]	Mean 4.17 (SD 0.68) on a scale of 1 to 6, where 1=awful, 2=poor, 3=okay, 4=good, 5=excellent, and 6=best imaginable
Razavi et al [31]	Mean 4.33 (SD 0.67) on a scale of 1 to 5 (“strongly agree” to “strongly disagree”)
Wargnier et al [24]	Pleasantness: mean 3.38 (SD 0.43); ease of following instructions: mean 3.38 (SD 0.47) on a scale of 1 to 4
Tokunaga et al [32]	Experimental questionnaire: 1=lowest, 4=highest; mean 3.58 (SD not given)
Tokunaga et al [19]	No quantitative usability data
Tsiourti et al [33]	Usability: mean 62.2 for Switzerland and mean 52 for the Netherlands on an unspecified scale (SD not provided)
Jegundo et al [34]	USE^a^ questionnaire from Lund [41]; 7-point Likert scale; total score 5.06 (SD 1.10) on a scale of 1 to 7
Oliveira et al [35]	No quantitative usability data

^a^USE: Usefulness, Satisfaction, and Ease of Use.

Other outcome data reported were *efficacy of nonverbal improvement* (1/8, 13%), *efficacy of verbal improvement* (1/8, 13%), *conversation quality* (1/8, 13%), *feasibility of interaction* (2/8, 25%), *observer usability assessment* (1/8, 13%), *critical incident registration* (1/8, 13%), and *speech recognition quality* (2/8, 25%). The reported values were difficult to combine at an aggregated level and to make them result in a general recommendation.

All agents found in this review were female characters, except for one. Studies from Western countries show *white-skinned* agents with varying hair colors and ages and a realistic look, whereas studies from Japan feature an anime-influenced character with a Japanese female look. These papers provide little information on facial muscle motions and lip synchronization when talking. Of the 8 studies, 5 (63%) showed mainly the head and shoulders of the agent, whereas the remaining 3 (37%) studies showed almost the complete agent body.

The duration of the interactions between the participant and the agent was relatively short, with a few turns per topic. These studies provide little information on the exact duration and how and when the interaction was stopped. These studies were concentrated in the United States (2/8, 25%), Europe (4/8, 50%), and Japan (2/8, 25%). No studies from China or Korea were found; however, this may be because of the selection of papers in the English language.

Reports on the cognitive status of the participants vary in the 8 studies. For 5 (63%) of the 8 studies, no related details were given, 1 (13%) study included participants with self-reported mild difficulties, 1 (13%) study included participants diagnosed with mild cognitive impairment, and 1 (13%) study conducted a Mini Mental State Examination among the participants. The latter study reported an average Mini Mental State Examination score of 22.9, consistent with mild dementia and a relevant target group for an intervention with a virtual agent.

According to our assessment, the quality of the studies varied between 0.58 and 0.83. Kmet et al [28] did not specify an absolute value for these outcomes, for example, in the sense that papers could be classified as having a good or bad methodological quality based on that value. Nevertheless, the overview in the study quality evaluation section of this paper enables comparison between the included studies. Although the study objectives and design were generally clear, only 1 (13%) of the 8 studies included a random allocation of participants to separate conditions [30]. The average number of participants in the studies (n=18) was rather low, and most articles seemed to focus more on the technical development of the system than on a thorough user evaluation. Moreover, verbal interactions were generally short. Most studies also lacked control of confounding parameters.

The results of this study, in terms of the number and scope of the studies found, were compared with the findings of relevant reviews mentioned in the *Introduction* section of this paper. The observation from Car et al [16] that there is a predominance of text-based conversational agents, with only a few apps using speech as the main mode of communication, remains valid. Although speech is considered a comfortable interaction modality for older adults, the difficulty of realizing free speech-based interaction with an agent is still present.

Xie et al [12] called for more systematic designs and evaluations of AI systems, and this is supported by the results herein showing a limited number of experimental studies targeting older adults with memory problems. Schachner et al [13] also found that the number of studies is scarce and mostly quasiexperimental, and Bevilacqua et al [15] concluded that the number of studies should increase.

For further research, it would be useful to evaluate IVAs, as shown in Figure 1, targeting people with other health conditions. For example, Laranjo et al [17] found 2 studies, one for military personnel with PTSS and one on training for people with autism spectrum disorders. Provoost et al [20] reviewed embodied conversation agents in clinical psychology, targeting individuals with autism, depression, anxiety, PTSS, schizophrenia, and substance abuse. Milne-Ives [22] discovered virtual agents for alcohol counseling, depression, and suicide prevention.

Regarding the design features of the virtual agent, Loveys et al [21] provided input for the design process by evaluating requirements for language use, behavior, emotional expression, embodiment, appearance, and personality. These findings may be considered when developing future virtual agents for people with amnesia.

### Limitations

This study has some limitations. Although the authors made an effort to screen the titles, abstracts, and papers carefully and applied a snowball method to identify additional studies by checking the references in selected papers, we do not exclude the possibility that some studies were overlooked. Second, in many studies, the actual implementation of the dialogue and the information exchange was difficult to assess. Third, many studies describe only the requirements or designs of virtual agents but provide no or very little information on the experiments conducted. Such studies were excluded, but we cannot rule out that, by doing so, relevant studies were missed. Fourth, *memory problems* is used in this paper as an umbrella term for the more formal terms dementia, Alzheimer, amnesia, or mild cognitive impairment, but was not used as an explicit search term, and this may have caused that a study was overlooked.

### Conclusions

Few studies have described actual experiments with IVAs in dialogue with older adults with memory problems. The dialogue contents are quite simple and superficial, especially on part of the participants, and often limited to only *yes* or *no*. More research is needed to develop real, useful, and prolonged dialogue between virtual agents and older adults. Another conclusion is that more research into the effectiveness of IVAs is needed, for example, through randomized controlled trials.

The reporting on the human-agent interaction characteristics often lacks many details, such as the exact contents of the dialogues, the starting and ending of the dialogue, and the graphical features of the avatar (static and dynamic). This makes it difficult to compare the experiments and to assess the status of the applied technology. A more standardized approach toward reporting human-agent interaction characteristics would be helpful for future research. Audio and video recordings of such interactions would provide even more information that will benefit the research community.

## Appendix

Multimedia Appendix 1 PRISMA (Preferred Reporting Item for Systematic Reviews and Meta-Analyses) checklist.

Multimedia Appendix 2 PRISMA (Preferred Reporting Item for Systematic Reviews and Meta-Analyses) abstract checklist.

## Abbreviations

AD: Alzheimer disease
AI: artificial intelligence
IVA: intelligent virtual agent
NLP: natural language processing
PRISMA: Preferred Reporting Items for Systematic Reviews and Meta-Analyses
PTSS: posttraumatic stress syndrome

## References

[1] O'Brien JT (1999). Age-associated memory impairment and related disorders. Adv Psychiatr Treat.

[2] Hugo J, Ganguli M (2014). Dementia and cognitive impairment: epidemiology, diagnosis, and treatment. Clin Geriatr Med.

[3] Vermunt L, Sikkes SA, van den Hout A, Handels R, Bos I, van der Flier WM, Kern S, Ousset PJ, Maruff P, Skoog I, Verhey FR, Freund-Levi Y, Tsolaki M, Wallin AK, Olde Rikkert M, Soininen H, Spiru L, Zetterberg H, Blennow K, Scheltens P, Muniz-Terrera G, Visser PJ, Alzheimer Disease Neuroimaging Initiative, AIBL Research Group, ICTUS/DSA study groups (2019). Duration of preclinical, prodromal, and dementia stages of Alzheimer's disease in relation to age, sex, and APOE genotype. Alzheimers Dement.

[4] Janssen O, Vos SJ, Handels R, Vermunt L, Verheij R, Verhey FR, van Hout H, Visser PJ, Joling KJ (2020). Duration of care trajectories in persons with dementia differs according to demographic and clinical characteristics. J Am Med Dir Assoc.

[5] Buchan J, Campbell J, Dhillon I, Charlesworth A (2019). Labour market change and the international mobility of health workers. Health Foundation.

[6] (2019). World Population Prospects 2019: Highlights (ST/ESA/SER.A/423). Department of Economic and Social Affairs, Population Division, United Nations.

[7] (2019). Proceedings of the 19th ACM International Conference on Intelligent Virtual Agents.

[8] Skantze G (2007). Error handling in spoken dialogue systems-managing uncertainty, grounding and miscommunication.

[9] Watkins I, Xie B (2014). eHealth literacy interventions for older adults: a systematic review of the literature. J Med Internet Res.

[10] (2019). Shaping Europe’s digital future - Assessment List for Trustworthy Artificial Intelligence (ALTAI) for self-assessment. European Commission.

[11] (2020). Shaping Europe’s digital future - Ethics guidelines for trustworthy AI. European Commission.

[12] Xie B, Tao C, Li J, Hilsabeck RC, Aguirre A (2020). Artificial intelligence for caregivers of persons with Alzheimer's disease and related dementias: systematic literature review. JMIR Med Inform.

[13] Schachner T, Keller R, V Wangenheim FV (2020). Artificial intelligence-based conversational agents for chronic conditions: systematic literature review. J Med Internet Res.

[14] Ienca M, Fabrice J, Elger B, Caon M, Scoccia Pappagallo A, Kressig RW, Wangmo T (2017). Intelligent assistive technology for Alzheimer's disease and other dementias: a systematic review. J Alzheimers Dis.

[15] Bevilacqua R, Casaccia S, Cortellessa G, Astell A, Lattanzio F, Corsonello A, D'Ascoli P, Paolini S, Di Rosa M, Rossi L, Maranesi E (2020). Coaching through technology: a systematic review into efficacy and effectiveness for the ageing population. Int J Environ Res Public Health.

[16] Tudor Car L, Dhinagaran DA, Kyaw BM, Kowatsch T, Joty S, Theng YL, Atun R (2020). Conversational agents in health care: scoping review and conceptual analysis. J Med Internet Res.

[17] Laranjo L, Dunn AG, Tong HL, Kocaballi AB, Chen J, Bashir R, Surian D, Gallego B, Magrabi F, Lau AY, Coiera E (2018). Conversational agents in healthcare: a systematic review. J Am Med Inform Assoc.

[18] King AC, Dwan C (2019). Electronic memory aids for people with dementia experiencing prospective memory loss: a review of empirical studies. Dementia (London).

[19] Tokunaga S, Horiuchi H, Takatsuka H, Saiki S, Matsumoto S, Nakamura M, Yasuda K (2016). Implementation and evaluation of interactive memory-aid agent service for people with dementia. Proceedings of the 7th International Conference on Digital Human Modeling: Applications in Health, Safety, Ergonomics and Risk Management.

[20] Provoost S, Lau HM, Ruwaard J, Riper H (2017). Embodied conversational agents in clinical psychology: a scoping review. J Med Internet Res.

[21] Loveys K, Sebaratnam G, Sagar M, Broadbent E (2020). The effect of design features on relationship quality with embodied conversational agents: a systematic review. Int J of Soc Robotics.

[22] Milne-Ives M, de Cock C, Lim E, Shehadeh MH, de Pennington N, Mole G, Normando E, Meinert E (2020). The effectiveness of artificial intelligence conversational agents in health care: systematic review. J Med Internet Res.

[23] Saredakis D, Szpak A, Birckhead B, Keage HA, Rizzo A, Loetscher T (2020). Factors associated with virtual reality sickness in head-mounted displays: a systematic review and meta-analysis. Front Hum Neurosci.

[24] Wargnier P, Benveniste S, Jouvelot P, Rigaud AS (2018). Usability assessment of interaction management support in LOUISE, an ECA-based user interface for elders with cognitive impairment. Technol Disabil.

[25] Deep learning. Wikipedia, the Free Encyclopedia.

[26] A brief history of speech recognition. Sonix.

[27] (2007). Publish or Perish. Anne-Wil Harzing.

[28] Kmet LM, Cook LS, Lee RC (2004). Standard quality assessment criteria for evaluating primary research papers from a variety of fields. Alberta Heritage Foundation for Medical Research.

[29] Page MJ, McKenzie JE, Bossuyt PM, Boutron I, Hoffmann TC, Mulrow CD, Shamseer L, Tetzlaff JM, Akl EA, Brennan SE, Chou R, Glanville J, Grimshaw JM, Hróbjartsson A, Lalu MM, Li T, Loder EW, Mayo-Wilson E, McDonald S, McGuinness LA, Stewart LA, Thomas J, Tricco AC, Welch VA, Whiting P, Moher D (2021). The PRISMA 2020 statement: an updated guideline for reporting systematic reviews. BMJ.

[30] Ali R, Hoque E, Duberstein P, Schubert L, Razavi SZ, Kane B, Silva C, Daks JS, Huang M, Van Orden K (2021). Aging and engaging: a pilot randomized controlled trial of an online conversational skills coach for older adults. Am J Geriatr Psychiatry.

[31] Razavi SZ, Schubert LK, Kane B, Ali MR, Van Orden K, Ma T (2019). Dialogue design and management for multi-session casual conversation with older adults. arXiv (forthcoming).

[32] Tokunaga S, Tamamizu K, Saiki S, Nakamura M, Yasuda K (2017). VirtualCareGiver: -ersonalized smart elderly care. Int J Softw Innov.

[33] Tsiourti C, Moussa MB, Quintas J, Loke B, Jochem I, Lopes JA, Konstantas D (2016). A virtual assistive companion for older adults: design implications for a real-world application. Proceedings of 2016 SAI Intelligent Systems Conference.

[34] Jegundo AL, Dantas C, Quintas J, Dutra J, Almeida AL, Caravau H, Rosa AF, Martins AI, Pacheco Rocha N (2020). Perceived usefulness, satisfaction, ease of use and potential of a virtual companion to support the care provision for older adults. Technologies.

[35] Oliveira F, Abad A, Trancoso I (2020). A multiplayer voice-enabled game platform for the elderly. Computational Processing of the Portuguese Language: 14th International Conference.

[36] Parsons TD, Schermerhorn P, Mcmahan T, Asbee J, Russo N (2017). An initial validation of virtual human administered neuropsychological assessments. Annu Rev CyberTher Telemed.

[37] Ujiro T, Tanaka H, Adachi H, Kazui H, Ikeda M, Kudo T, Nakamura S (2018). Detection of Dementia from Responses to Atypical Questions Asked by Embodied Conversational Agents. Proc Interspeech.

[38] Walker G, Morris LA, Christensen H, Mirheidari B, Reuber M, Blackburn DJ (2021). Characterising spoken responses to an intelligent virtual agent by persons with mild cognitive impairment. Clin Linguist Phon.

[39] Sidner CL, Bickmore T, Nooraie B, Rich C, Ring L, Shayganfar M, Vardoulakis LM (2018). Creating new technologies for companionable agents to support isolated older adults. ACM Trans Interact Intell Syst.

[40] Sauro J, Lewis J (2016). Quantifying the User Experience - Practical Statistics for User Research. 2nd edition.

[41] Lund AM (2001). Measuring usability with the USE questionnaire. Usability Interface.

